# Prolonged 59‐day course of COVID‐19: The case of a SARS‐CoV‐2 shedding persistency in a healthcare provider

**DOI:** 10.1002/ccr3.3547

**Published:** 2020-11-28

**Authors:** Adeline Penchenat, Zarrin Alavi, Christopher Payan, Jacques‐Olivier Pers

**Affiliations:** ^1^ Odontology CHRU de Brest Brest France; ^2^ Inserm CIC 1412 CHRU de Brest Brest France; ^3^ Bacteriology‐Virology Hospital Hygiene and Parasitology‐Mycology CHRU de Brest Brest France; ^4^ LBAI, U1227 Univ Brest Inserm Brest France

**Keywords:** COVID‐19, healthcare provider, SARS‐Cov‐2 persistency

## Abstract

For health professionals, repeated SARS‐CoV‐2 RNA detection through PCR combined with properly validated serologic assays for the presence of anti‐IgM and anti‐IgG antibodies should be mandatory.

## Graphical abstract

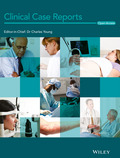

## 1 INTRODUCTION

Since December 2019, epidemiological and clinical characteristics of patients with COVID‐19 are being reported worldwide. A recent study of 191 Chinese patients reported a median viral shedding duration of 20.0 days (IQR 17.0‐24.0) in survivors. However, SARS‐CoV‐2 was detectable until death in nonsurvivors. Moreover, 37 days were reported to be the longest observed duration of viral shedding in survivors.^1^

We report the case of a 25 years old woman, working as a resident at the dental clinic of Brest University Hospital, who developed the symptoms of COVID‐19 and was tested positive for SARS‐CoV‐2 RNA twice during 53 days.

## 2 METHODS

A summary of patient's history, family history, environment, background, clinical examination, biological data, diagnosis, and treatments of the clinical case is provided in Table 1.

**Table 1:** Family history, environment, background, patient's history, clinical examination, biological data, diagnosis, and treatments of the clinical case

Family history, family environment, background history	Patient's history	Clinical examination	Biological data	Diagnosis	Treatments
‐ There is no medical history in the family except an history of juvenile rheumatoid arthritis in a sister of the patient ‐ The patient lives in Brest for 2 y (northwest of France) and her family lives in the southwest of France in the countryside ‐ On 14 March‐15 March 2020 the family gathered in eastern France for a family weekend ‐ On March 14, a patient'sister had a fever during one day and nothing else ‐ On March 19, another pregnant sister was tested positive SARS‐CoV‐2 RNA PCR ‐ On March 17, the patient's parents had flu symptoms but were not tested. For both, serologic ELISA test for IgG antibodies to SARS‐COV‐2 was positive on May 2020 ‐All of them were placed in quarantine at home during the symptomatic period	‐ The patient was previously in good health, non‐smoker, without any medication or known risk factor for COVID‐19 ‐ She only reported a history of psoriasis of the hand, elbow, and scalp once a year in the past 10 y	‐ On 15 March 2020, during the symptomatic period, she presented digestive disorders, diarrhea, rhinitis, rheum, buccal aphthous, dry cough, and a 38°C fever associated with chills, high fatigue, ageusia, a worsening (upon ante flexion) headache, localized at the forehead and the temporal region, body aches especially in the cervical region, the upper back, and the legs during the first week, cutaneous manifestations (pruritic lesions) covered all her limbs with approximately 10 x 2mm of red papules, dry cough gradually turned into a wet cough during the second week ‐ Asymptomatic on April 2 ‐ She goes back to work on April 6 ‐ On May 6, she presented with rhinitis, flu symptoms, and great fatigue along with a dry cough, nausea, ageusia, and eruption of erythematosus red papules on the hand ‐ On May 12, 59 d after the appearance of the first set of symptoms of COVID‐19, once again, she became asymptomatic with a negative SARS‐CoV‐2 RNA PCR	‐ First positive SARS‐CoV‐2 RNA PCR on March 18 ‐ On March 29, a negative serologic test with colloidal gold particles for detection of anti‐SARS‐CoV‐2 IgM and IgG ‐On May 9, she is tested positive for SARS‐CoV‐2 RNA on quantitative PCR for the second time ‐On May 12, a serologic ELISA test for IgG antibodies to SARS‐COV‐2 was positive with an index (signal‐to‐cutoff ratio) of 5 with a positive SARS‐CoV‐2 cutoff of 1.1 (Mean:10)	‐ The diagnosis of COVID‐19 was given on a first positive SARS‐CoV‐2 RNA PCR on March 18 ‐ The diagnosis of COVID‐19 was given on a second positive SARS‐CoV‐2 RNA PCR on May 9	‐ Only a treatment for symptoms was given to reduce pain such as paracetamol

## 3 RESULTS

On 15 March 2020, this young lady presented with the first phase of COVID‐19 symptoms, that is, digestive disorders, diarrhea, rhinitis, rheum, buccal aphthous, dry cough, and a 38°C fever. She was previously in good health, non‐smoker, without any medication, or known risk factor for COVID‐19. She only reported a history of psoriasis of the hand, elbow, and scalp once a year in the past 10 years.

Given her healthcare provider status and her mild symptoms, after a first positive SARS‐CoV‐2 RNA PCR on March 18, she was placed in quarantine at her home. This symptomatic period was associated with appearance of chills, fever (38°C), high fatigue, and ageusia. In addition, she presented with a worsening (upon ante flexion) headache, localized at the forehead and the temporal region. She also presented with body aches especially in the cervical region, the upper back, and the legs during the first week. Additionally, cutaneous manifestations (pruritic lesions) covered all her limbs with approximately 10 × 2 mm of red papules. Her dry cough gradually turned into a wet cough during the second week.

She became asymptomatic on April 2, and despite a negative serologic test with colloidal gold particles for detection of anti‐SARS‐CoV‐2 IgM and IgG on March 29, she was allowed to go back to work on April 6th.

In brief, she became asymptomatic during 5 weeks of convalescence. On May 6, she presented with rhinitis, flu symptoms, and great fatigue along with a dry cough, nausea, ageusia, and eruption of erythematosus red papules on the hand. On May 9, she tested positive for SARS‐CoV‐2 RNA on quantitative PCR for the second time. Of note, other dental residents showed negative PCR results.

On May 12, 59 days after the appearance of the first set of symptoms of COVID‐19, once again, she became asymptomatic with a negative PCR. In addition, a serologic ELISA test for IgG antibodies to SARS‐COV‐2 was positive with an index (signal‐to‐cutoff ratio) of 5 with a positive SARS‐CoV‐2 cutoff of 1.1. Of note, on August 31, the serologic ELISA test for IgG antibodies to SARS‐COV‐2 was negative.

## 4 DISCUSSION

Our case presents useful data on a possible clinical course of this novel illness including viral shedding: incubation period, contagious phase, and stages of a mild COVID‐19.

In the absence of nasopharyngeal swab during the first convalescence period, the recurrence of the symptoms along with the second positive (SARS‐CoV‐2 RNA) PCR after the first convalescence period could convey two hypotheses: either reactivation or persistence of SARS‐CoV‐2 shedding. To date, only a 33‐year‐old man from Hong Kong was reported to have the first confirmed case of COVID‐19 reinfection.^2^ In this very recent case, 24 nucleotides differed between the viruses from the first and second infection episode. These were amino acid differences in nine proteins, including a 58 amino acid truncation of ORF8 protein that was present only in the virus from the first infection. The latter findings suggest that acquired immunity after natural infection may be short lived. In our presented case, re‐infection hypothesis seemed unlikely according to the following. The reported COVID‐19 infection rate in our region was low with no new COVID‐19‐positive patients during the previous 8 days before her relapse.

Furthermore, according to the Guangdong COVID‐19 surveillance system on epidemiology and characteristics of re‐positive cases between 23 January and 26 February 2020,^3^ the percentage of SARS‐CoV‐2 re‐positive cases was around 14%. All re‐positive cases in the Guangdong study developed only mild or moderate symptoms on initial diagnosis, with the median age being significantly lower than that of the general COVID‐19 cases. Furthermore, according to the WHO public health surveillance guidance for COVID‐19,^4^ a robust neutralizing antibody response and potential virus genome degradation are detected in almost all re‐positive cases, suggesting a substantially lower transmission risk, especially through respiratory routes. In our case report, although the symptoms were mild, the neutralizing antibody response was weak.

Our case presents the first reported COVID‐19 healthcare provider with prolonged viral shedding due to either disease reactivation or persistence^5^ in Europe. To date, this is one of the longest reported prolonged viral shedding, that is, a 59‐day course of the disease. Consistent with our case report, six cases of healthcare professionals in Brazil who recovered but again presented symptoms consistent with COVID‐19, with new positive RT‐PCR test results were also recently reported.^6^

## 5 CONCLUSION

Clinicians should be alerted on the rationale for a close monitoring of viral load and acquired immunity and a longer than recommended 15 days of isolation strategy in such subgroup of COVID‐19 patients.

## CONFLICT OF INTEREST

None declared.

## AUTHOR CONTRIBUTIONS

All the authors have been involved in the work leading to the publication of the paper, contributed to the writing of the manuscript, and approved it.

## Data Availability

The data that support the findings of this study are available on request from the corresponding author. The data are not publicly available due to privacy or ethical restrictions.
